# Pain and Pain Management in Sea Turtle and Herpetological Medicine: State of the Art

**DOI:** 10.3390/ani12060697

**Published:** 2022-03-10

**Authors:** Ilenia Serinelli, Simona Soloperto, Olimpia R. Lai

**Affiliations:** 1 Independent Researcher, 73100 Lecce, Italy; i.serinelli89@gmail.com; 2 Sea Turtles Rescue Center “Luigi Cantoro” Torre Guaceto Protected Area, 72012 Carovigno, Italy; solsimona@libero.it; 3 Department of Veterinary Medicine, University of Bari “Aldo Moro”, 70010 Valenzano, Italy

**Keywords:** pain management, analgesia, sea turtles, chelonians, reptiles, NSAIDs, opiates

## Abstract

**Simple Summary:**

Rescue and rehabilitative medicine of sea turtles must deal with several circumstances that would be certainly considered painful in other species (trauma, situations that require surgery); thus, it would be natural to consider the use of analgesic drugs to manage the pain and avoid its deleterious systemic effects to guarantee a rapid recovery and release. However, in these animals (as well as in reptiles in general), many obstacles stand in the way of the application of safe and effective therapeutic protocols. It has been demonstrated that, anatomically and physiologically, turtles and reptiles in general must be considered able to experience pain in its definition of an “unpleasant sensory and emotional experience”. Unfortunately, specific studies concerning sea turtles and reptiles on pain assessment, safety, and clinical efficacy of analgesic drugs currently in use (mostly opioids and non-steroidal anti-inflammatory drugs—NSAIDs) are scarce and fragmentary and suffer from some basic gaps or methodological bias that prevent a correct interpretation of the results. At present, the general understanding of the physiology of reptiles’ pain and the possibility of its reasonable treatment is still in its infancy, considering the enormous amount of information still needed, and the use of analgesic drugs is still anecdotal or dangerously inferred from other species.

**Abstract:**

In sea turtle rescue and rehabilitative medicine, many of the casualties suffer from occurrences that would be considered painful in other species; therefore, the use of analgesic drugs should be ethically mandatory to manage the pain and avoid its deleterious systemic effects to guarantee a rapid recovery and release. Nonetheless, pain assessment and management are particularly challenging in reptilians and chelonians. The available scientific literature demonstrates that, anatomically, biochemically, and physiologically, the central nervous system of reptiles and chelonians is to be considered functionally comparable to that of mammals albeit less sophisticated; therefore, reptiles can experience not only nociception but also “pain” in its definition of an unpleasant sensory and emotional experience. Hence, despite the necessity of appropriate pain management plans, the available literature on pain assessment and clinical efficacy of analgesic drugs currently in use (prevalently opioids and NSAIDs) is fragmented and suffers from some basic gaps or methodological bias that prevent a correct interpretation of the results. At present, the general understanding of the physiology of reptiles’ pain and the possibility of its reasonable treatment is still in its infancy, considering the enormous amount of information still needed, and the use of analgesic drugs is still anecdotal or dangerously inferred from other species.

## 1. Introduction

The need to treat pain is particularly felt in the rehabilitation medicine of sea turtles, considering that the situations for intervention on stranded turtles are certainly and in high percentages to be considered painful: lesions of the gastrointestinal tract associated with ingestion of hooks and monofilament lines, postoperative pain in surgeries required for the removal of gastrointestinal foreign bodies, limb injuries after entanglement in derelict fishing gear, amputation of limbs, fractures and excoriations due to impact with the rocks of animals in cold stunning, boat strikes, and reconstruction of carapace fractures.

Recognizing and managing pain in animals is currently considered clinically and ethically essential in veterinary practice, although little attention was paid to the issue in the past, especially in wild species or exotics. The underlying logic may have been quite heterogeneous; ideological obstacles (for example, the idea that pain is a “natural” response, or that animals do not perceive it), difficulty in recognizing and quantifying it, unawareness of its deleterious effects on the recovery of the animal, fear of side-effects and the possible toxicity of the drugs, and lack of familiarity with therapeutic protocols may all have contributed to making analgesia not practiced in reptiles and wild species in particular.

With an anthropocentric point of view, “pain” infers a higher cortical level of information processing, which is considered a characteristic of *Homo sapiens*, and human pain management is referred as “analgesia”, while “nociception” and “antinociception” should be used when dealing with mammalian and nonmammalian species [1]. Whether nonmammalian species such as reptiles possess the anatomical and physiological structures to differentiate “nociception” from “pain” and the “reflex” response to a noxious stimulus from “experiencing pain” has long been a subject of controversy [2,3,4].

Nociception has a conservative value and, as such, this tool is a sensory ability that arose very early in the evolution of life and is also present in very simple organisms. In most animal species, it is possible to describe defensive/protective somatic reactions, typically urgent and primordial, in response to an unpleasant sensory experience that is defined as “pain”. Two series of physiological integrating events characterize this complex behavioral phenomenon, widespread in an almost universally but extremely diversified way in the animal kingdom. The first set of events regulates the sensory and objective components of the transmission of pain sensation (nociception) that promote a rapid reflex of avoidance or withdrawal from the noxious source. The second set represents the subjective component of pain perception and processing that originate the individual response to the noxious sensory experience [5]. Pain represents a physiologically and psychologically complex entity, defined by the International Association for the Study of Pain [6] as “an unpleasant sensory and emotional experience associated with, or resembling that associated with, actual or potential tissue damage”, thus requires a multidisciplinary approach for its understanding and management [7]. Pain is classically defined as “an aversive sensory experience caused by actual or potential injury that elicits protective and vegetative reactions, results in learned behavior, and may modify species specific behavior” [8], following which “animals in pain quickly learn to avoid the noxious stimulus and demonstrate sustained changes in behavior that have a protective function to reduce further injury and pain, prevent the injury from recurring, and promote healing and recovery” [7]. Associating the adverse experience with suffering quickly teaches the individual to avoid the dangerous situation and, in this way, becomes an adaptive survival mechanism in the hostile environment [6,7,8]. At the present level of our knowledge, it is not possible to describe qualitatively and quantitatively the experience of pain in reptiles, but it is certain that they can experience noxious stimuli as part of an unpleasant event and are able to modify their behavior in a way to avoid this situation. Probably a more inclusive definition of pain in animals would be “a sensory experience representing awareness of damage or the potential for tissue damage that results in a behavioral and physiologic response to minimize/prevent the recurrence and promote healing of damage”, suggesting that animals can surely experience a painful sensation like humans, but human and animal pain can certainly be different, at least from a qualitative and quantitative point of view [9,10].

Criteria are needed to differentiate nociception from pain; rapidly withdrawing from the source of the pain stimulus is a reflex and does not require emotional processing. The criteria for defining a species as capable of experiencing pain are indicated as (1) possession of peripheral sensory receptors or “nociceptors”, (2) pathways from nociceptors to the spinal cord, (3) initial processing of the noxious stimuli in the spinal dorsal horn, (4) ascending pathways to the brain, (5) brain structures such as the human cortex processing the incoming stimuli, (6) descending pathways to control the defensive responses, (7) endogenous opioids and their receptors in a nociceptive neural system, (8) learning of behaviors that preserve the painful experience from the future repetition, and (9) reduction in pain-related attitudes (sign of discomfort) after administration of analgesics [2,3,4,5,6,7]. Therefore, if the animal species has the neural structure to sense the damage and report it to the central nervous system, adapting the behavior accordingly, as a human being would, it should be treated humanely [10,11]. The inability of animals to communicate pain and the human inability to understand how species other than humans emotionally experience pain cannot make us ignore the fact that all potential or actual tissue damage must also be considered painful in animals [6]. Contemporary, it is of the most importance to note that the absence of clinically evident signs of pain is not an indication of the absence of pain in wild species such as reptiles [12].

In the present paper, the available literature data on anatomy, physiology, pharmacology, and therapeutics of pain management in sea turtles (or, more generically, on chelonians or reptiles, when specific information on sea turtles is lacking) were collected and critically analyzed, reporting the “state of the art” in this topic.

## 2. Anatomy of Pain

The succession of events at the basis of pain originates from a noxious stimulus (mechanical, thermal, chemical) that could potentially or actually damages the tissues; this activates specialized sensors (nociceptors) and afferent pain pathways (nociceptive neurons) which activate the central nociceptive system via synaptic mechanisms. This system consists of the neurons of the spinal cord and the thalamo-cortical system located in phylogenetically ancient areas of the brain, preserved in nonmammalian vertebrates, such as the limbic system, the thalamus, the hypothalamus, and the central part of the reticular formation of the encephalic trunk. Collectively, these structures are responsible for the perception, memorization, and emotional processing of the response to the pain sensation [5].

Pathways required for the brain to process pain include (1) transduction, (2) transmission, (3) modulation, (4) projection, and (5) perception [4].

### 2.1. Transduction

The existence of nociceptive mechanisms that respond to noxious stimuli has been demonstrated in mammals, as well as in nonmammalian vertebrates; in the latter, opioid peptidergic neuronal pathways have also been described, which represent the mechanisms of neuromodulation of pain sensitivity [5].

All living organisms share the ability to recognize and respond adequately to situations that are potentially dangerous for their survival. Multicellular organisms have evolved peripheral nerve endings that distinguish harmless from harmful stimuli, decoding and processing potentially or actual harmful stimuli. Nociceptors are specific receptors of primary or first-order sensory afferent neurons, defined as a distinct class of nerve fibers that conduct noxious information [13]. They are highly conserved across many phyla, from invertebrates to mammals, including reptiles [2], and they can be activated by a milieu of intense and potentially noxious external stimuli capable of depolarizing these “high-threshold” fibers. Reptiles have large terminal expansions of unencapsulated nerve endings at the integumentary level (intraepidermal nociceptors), whose variable morphology characterizes the different sensitivities (touch, thermal, and mechanical) as demonstrated in the trigeminal ganglia of crotaline snakes [14].

Usually, nociceptors are multimodal tools found in the neural systems of vertebrates and invertebrates, responding to various noxious or potentially noxious stresses, such as intense mechanical stimuli, extreme temperatures, and chemical stimuli [15]. For example, the high threshold cationic channel receptors (Na^+^ and Ca^2+^) TRPV1 (transient receptor potential vanilloid 1) are activated by temperatures above 43 °C but also by vanilloid ligands such as capsaicin and by conditions of acid tissue pH [16]. Other nociceptors can function in a monomodal mode, such as mechanoreceptors (intense pressure, swelling, tissue damage), chemoreceptors, endogenous chemical stimuli such as mediators of inflammation, or exogenous chemical stimuli such as capsaicin, menthol, and formalin [15]. Nociceptive terminals can also respond to chemical stimuli like those induced by inflammatory mediators, which can trigger the painful sensation directly or act as sensitizers by lowering the excitation threshold of the nociceptive terminals themselves (bradykinin, histamine, prostaglandin E2 (PGE2), nerve growth factor (NGF)), giving rise to hyperalgesia and allodynia [5].

### 2.2. Transmission

After the transduction of the stimulation, the information travels to the dorsal horn of the spinal cord through small caliber fibers, myelinated (A fibers) or nonmyelinated (C fibers) at different conduction speeds. The “fast” fibers (15 m/s) Aβ are activated by harmless stimuli of low intensity, while the Aδ fibers are responsible for the conduction of acute sharp “first pain” that is sensed immediately after the noxious stimulus; the “second pain”, dull, burning, poorly localized, and more persistent (tissue damage, inflammation, chronic pain), is conducted by the “slow” (1 m/s) polymodal C fibers. Polymodal and bimodal nociceptors, with projections of Aδ and C fibers at the spinal cord dorsal horn and trigeminal, have been found in fish, amphibians, reptiles, birds, and mammals [15].

In the spinal cord, there is the first synaptic transmission between the first- and second-order afferent neurons, effected by glutamate which mediates a rapid excitatory transmission on postsynaptic receptors NMDA (voltage-dependent influence of Na^+^ and Ca^2+^) and AMPA (influence of Na^+^), along with neurokinins such as substance P. Transmission is mediated by several neurotransmitters, including substance P, glutamatergic exciters, and γ-aminobutyric acid-GABA-ergic inhibitors, which have also been found in reptiles [17]. The substance P system is highly conserved phylogenetically and is also present in reptiles, found in *Trachemys dorbigni* [18], *Chrysemys picta picta*, and *Trachemys scripta elegans* [19]. Ascending pathways of pain transmission (spinothalamic projections analogous to the neospinothalamic traits of “fast pain” and paleospinothalamic traits of “slow pain” in mammals have been identified in reptiles, as well as the trigeminal trait [9].

### 2.3. Modulation

Inhibitory modulation of pain transmission at the spinal level, before projection to the brain, is guaranteed by the “gate control” mechanism, according to which small interneurons with GABAergic or enkephalinergic transmission coordinate the painful information coming from the periphery and descendent pain inhibitory system of the nociceptive neuronal system. Non-algic stimuli (nonharmful mechanical afferents) also contribute to this control, which cause stimulation of Aβ low-threshold receptors and reduction of the output on Aδ and C afferent nerve fibers, which in turn inhibit transmission of signal to second-order neurons through gating at the *substantia gelatinosa* in the dorsal root ganglion of the spinal cord, preventing nervous stimuli from reaching the thalamus and cerebral cortex [20]. Interneurons have been identified in the spinal cord of red-eared slider *Trachemys scripta elegans*, in the gray matter that modulates limb movement [21,22].

In mammals, the descending inhibitory modulation of the nociceptive response is carried out by endogenous opiates; their receptors are characteristic of the periaqueductal gray, rostroventral medial medulla and dorsal horn of the spinal cord in mammals but have also been found in the central nervous system (CNS) of fish, amphibians, reptiles, and birds [15]. 

The presence of spinal projections originating in the brainstem (nucleus raphes inferior) reaching the superficial layers of the medullary dorsal horn was found in tokai gecko *Gekko gecko*, suggesting the presence of structures and mechanisms that mediate the descending inhibition of nociception as in mammals [23].

The study of the evolution of opioid peptides and nociceptin/orphanin and their receptors has led to researchers postulating the existence of one opioid precursor gene and one receptor gene in the vertebrate predecessor. The opiates precursor gene would have generated prepropeptides for endorphin (POMC), enkephalins, dynorphins, and nociceptin, while the ancestral receptor gene would have led to the δ, κ, μ, and nociceptin/orphanin receptors [24]. Endogenous opioids and other nociceptive neuropeptides (glutamate, substance P, calcitonin gene-related peptide) have been described in reptiles, although their precise function has not been determined [9].

Opioid peptides have been found in the nervous system of a wide range of nonmammalian species, from planarians to birds. Studies in RIA or HPLC have found them in amphibians as in earthworms. Since the three distinct families of opioid peptides seem to have appeared before the invertebrate–vertebrate divergence, it is not difficult to understand how endorphins, enkephalins, and dynorphins are all present in the CNS of reptiles [25]. *Trachemys scripta* isolated brainstem preparations have been used for the study of δ, κ, and μ receptors for opioids and their action in modulation/depression of respiration [26,27], as well as for the study of subtype α5 of GABA(A) receptors-α5GABA(A) [28]. The presence of endorphins has been demonstrated in the nervous system of reptiles by biochemical and immunohistochemical studies carried out in the green anole *Anolis carolinensis* [29], while dynorphins have been demonstrated by immunohistochemistry and with antisera that do not cross-react with the enkephalins in *Trachemys scripta elegans* [30]. In a study carried out on the CNS of *Chrysemys* and *Pseudemys* [25], the distribution of peptides in the group derived from the forebrain (or of very similar molecules capable of reacting with specific antibodies in the mammalian forebrain) was very similar to that described in mammals, with widespread localizations in various neurons and fibers of the CNS. At the telencephalic level, all reptiles have higher levels of enkephalinergic fibers and neurons. The enkephalins are more abundant in the basal ganglia than in the overlying telencephalic regions. Within the basal nuclei, enkephalins are present in striatal fibers and neurons, as well as in pale fibers, thus suggesting the existence of striatopallidal enkephalinergic projections. The connecting hypothalamic sensory nuclei generally have a scarcity of encephalic fibers, while the hypothalamus itself is very rich in neurons and enkephalinergic fibers, as are the gray matter of the midbrain, the trigeminal nucleus, and the dorsal fibers of the spinal cord. On this basis, the authors [25] state that (1) the molecular structure and distribution of neuropeptides (including substance P, somatostatin, and neurotensin) is fairly uniform among amniotes, suggesting a high evolutionary conservativeness, (2) the forebrain of reptiles is structurally very similar to that of mammals and originates the same enkephalinergic peptides, (3) the enkephalins of reptiles show the same localizations found in the CNS of birds and mammals, (4) many of the main distribution characteristics of enkephalins in birds and mammals were already present in the reptilian ancestors, (5) enkephalins reasonably exert their physiological effects in turtles on the same types of receptors present in birds and mammals, and (6) μ- and δ-type opioid receptors have been reported in reptiles, making it reasonable that enkephalins play a functional role in reptiles as in many of the same avian and mammalian districts, and this role is probably mediated by similar synaptic events [25].

### 2.4. Projection

The projection of sensory information to the cerebral cortex is conducted through the spinal white matter, originating from neurons present in the gray part, to the upper brain centers, including the thalamus, reticular system, and midbrain (spinothalamic, spinoreticular, and spinomesencephalic pathways). Structures like those present in mammals are also present in reptiles, albeit with minor differentiations at the level of the thalamus and pallium, described in pond slider *Trachemys scripta* [31], tokai gecko *Gekko gecko*, green iguana *Iguana iguana* [32], and Iberian wall lizard *Podarcis hispanicus* [33].

### 2.5. Perception

Perception includes the integration, processing, and recognition of sensory information at the level of brain, which coordinates the response to the noxious stimulus [4].

Neuroimaging in mammals made it possible to identify a “pain matrix” activated by stimuli from the spinothalamic and spinoreticular pathways. Although there is also evidence of areas corresponding to the amygdala in fish, homologous features in amphibians, reptiles, and birds are still poorly understood [14]. Comparative studies of brain structure and development found a common basic functional organization. In all vertebrates, behavior is modulated by common brainstem neuromodulatory circuits, such as the serotoninergic system. The *Reptilia* class includes more than 11,000 species, divided into different orders (*Testudinata*, *Rhynchocephalia*, *Squamata*, *Crocodylia*), which share common evolutionary, anatomical, functional, and developmental characters common to all vertebrates, with some morphological similarities of the embryonic stages (“phylotypic stage”) despite the differences of the adults. Among these, the structure and subdivisions of the brain show how all regions in the mammalian brain, including the cortex, have homologs in reptiles. The cortex is in fact a part of the pallium, a subdivision of the telencephalon which is preserved in all vertebrates [34]; during embryonic development, the subdivision of the pallium into dorsal (which forms the neocortex of mammals), ventral, lateral, and medial is the same for all species, despite the differences as adults. In addition, reptiles and mammals share a markedly distinct cortical structure, with common excitatory and inhibitory neurotransmitters [17]. All amniotes possess the dorsal pallium, with similar cell types and neuronal connections between different taxa. The pallium of reptiles has the dorsal cortex, the dorsal ventricular ridge, the olfactory region, the hippocampus, and the amygdala, i.e., the “emotional” portion of the brain [35]. The central processing may not be the same in evolutionarily distant species; however, the constant presence of nociceptive pathways, central processing, and descending inhibitory modulation by opioids suggests the presence of a common scheme for the decoding and integration of the pain stimulus at the level of CNS [15].

Therefore, anatomically, the reptilian brain possesses the structures necessary for the experience of “pain” and the connections between the spinal cord and the brainstem/dorsal thalamus in the midbrain, as well as the thalamocortical connections [36]; hence, there are no arguments to rule out this function in reptiles. However, questions such as “how” reptiles perceive pain and what nociception means for physiological homeostasis are still far from being answered, just as the quality of the pain experience is unknown [9,10].

## 3. Pain Assessment and Animal Models of Pain

On the basis of the characteristics of location, onset, duration, and etiopathogenesis, pain can be classified into different forms: somatic or visceral, acute or chronic, and physiological (acute inflammatory) or clinical (chronic inflammatory and neuropathic) nociceptive pain. Physiological pain is protective, well localized, and proportionate to the peripheral stimulus; furthermore, it disappears once the inflammatory process resolves. Clinical pain is triggered by significant trauma or inflammation of the tissues; it is pathological and debilitating, widespread, disproportionate to the peripheral stimulus, and it can continue beyond the resolution of the inflammatory process. Spontaneous, neuropathic or dysnociceptive pain, or modification of algic sensitivity in the case of hyperalgesia (mild pain perceived as intense) and allodynia (harmless stimulus perceived as noxious) represent alterations in the physiology of the conduction pathways of pain sensitivity and are difficult to treat pharmacologically [37]. While a great deal of research has been conducted on acute pain, there is still a paucity of data regarding chronic pain in herpetological medicine.

Pain recognition and assessment have always been an integral part of animal care and veterinary clinical practice, but clinical research on this aspect, especially with regard to animal welfare, has grown noticeably over the past 20 years. The concept of painful behavior indicates that pain, in fact, influences behavior-determining variations, and that the extent of behavioral changes is correlated with the severity of the painful experience.

Assessing pain in nonverbal species is an arduous challenge, and reptiles are among the most poorly understood and understandable species in this respect; however, apart from the anatomical and physiological identification in reptiles of the same or analogous structures responsible for the pathways of pain in mammals, some considerations lead to the consideration of reptiles as capable of feeling pain: the presence of behaviors evoked as a response to a pain stimulus, although often complex to interpret, especially in response to chronic pain, and the possibility of pharmacological modulation of the pain response (at least in some species) [38]. Despite this, in a study conducted among members of the Association of Reptile and Amphibian Veterinarians, 98% of respondents said they believe reptiles feel pain, but only 39% of them reported using pain relievers in more than 50% of cases [39]. The reasons for not using analgesic drugs were traced back to the inability to recognize the manifestations of pain, the lack of data on therapeutic efficacy, the concern about possible negative effects, and the absence of data on doses and posology to follow. Conversely, if not treated adequately, pain can cause various organic alterations (increased heart and respiratory rhythm, hypercapnia, increased protein catabolism, delay in repairing tissue damage, and prolonging the time necessary for complete recovery), particularly negative events in the case of wild animals [40,41].

Animal models of pain should provide the basis for understanding the pathophysiological mechanisms and for evaluating the therapeutic applicability of new drugs, where their predictability is a major issue. Reliably, models should produce behavioral indices related to pain (hyperalgesia and allodynia included), have a similar course to that observed in clinical practice, and possibly be applicable to different species, as receptor or neurotransmitter differences may occur [42].

The measurement of “pain sensation” in animals is mostly indirect, as there are no systems to test the “quality” of pain itself with current pain models. Apart from the “aversive” behaviors toward the noxious stimulus (vocalization, altered behaviors concerning the limb being stimulated), all measurements are based upon the evaluation of defense stimuli. The most used noxious reflex, in both acute (“phasic pain”) and chronic (“tonic pain”) pain models, is certainly the withdrawal time from the noxious stimulus, usually heat (45–52 °C) or compression. With this method, the analgesic or hyperalgesic effects of drugs in homeotherms, as well as the onset of allodynia (via von Frey filaments), were also tested [42,43].

Basic research on chronic pain in animals has also evolved considerably. Immediately after the introduction of the formalin test [44], the first nociceptive “tonic” test, it was hypothesized that the type of pain evoked was qualitatively different from the acute one, providing anatomical and neurochemical evidence of the dissociation of neural mediation in acute and chronic pain [45]. Since chronic pain, linked to tissue and nerve injuries, is the most clinically relevant, researchers’ attention is now turned to this type of assessment, using tests based on models of chronic tissue damage (carrageenan, zymosan, Freund’s complete adjuvant, mustard oil) but with still similar evaluation tests (paw-pressure test, paw-withdrawal test, acetone drop test) since they are thought to be easy to carry out, repeatable, and quantifiable [46].

Animal pain models are broadly divided into somatic and visceral pain models.

### 3.1. Somatic Pain Patterns

Acute pain models: they measure the nocifensive response of animals to a noxious stimulus, which is generally heat. This type of model has been used to evaluate the analgesic response to opioid drugs, which modify defense behavior, but do not detect the analgesic action of NSAIDs, which interact with mechanisms that are triggered during inflammatory pain. Using the thermal threshold test may prevent the detection of potentially clinically relevant analgesic actions by drugs [42].

Models of pathological, chronic inflammatory, and neuropathic pain: theoretically, the study of pain models that alter the physiological pain threshold (hyperalgesia) should lead to the understanding of drugs useful in the management of chronic pain. The models classically used are those of persistent pain induced by formalin inoculation (which induces spontaneous noxious behavior that is easy to assess [47]) and capsaicin. Moderate but continuous pain generated by tissue lesions is markedly different from acute but short-lived pain induced by above-threshold stimuli [47]. More sophisticated than acute pain, these models induce hyperalgesia through subcutaneous inoculation of inflammatory agents or intense UV radiation that induces a state of allodynia used as an assessment of inflammatory pain associated with first- and second-degree burns [42]. Moreover, the pain generated by nerve lesions (neuropathic), particularly resistant to analgesic treatments (e.g., with NSAIDs), has been reproduced with numerous techniques of direct neuronal damage at the peripheral level (Bennet: ligation of the sciatic nerve; Seltzer: partial ligation of the sciatic nerve; Chung: ligation of one of the two sciatic spinal emergencies [42,48,49]) and used to evaluate possible drugs for the management of neuropathic pain.

### 3.2. Visceral Pain Patterns

Visceral organs are highly sensitive to mechanical stimuli (distension, traction), ischemia, and inflammation, all of which evoke visceral pain. Among the main animal models that have been developed, there are specific systems of distension of hollow organs or of the capsule of parenchymatous organs (insertion of small balloons that can be inflated), evoking quantifiable responses such as contraction of the abdominal and pelvic muscles (evaluated by electromyography) or an increase in blood pressure and heart rate (evaluated by surgical implantation of intravenous catheters). Inflammatory pain is also evoked by injecting irritants (2,4,6-trinitrobenzenesulfonic acid (TNBS) diluted in alcohol, acetic acid, zymosan, acrolin, or cyclophosphamide) directly into the esophagus, ileus, colon, or urinary bladder, and the pain response is evaluated with electromyography or analgesiometry [50].

### 3.3. Limits in the Application of Pain Models in Reptiles

In all animal models, it remains unclear which indicator should be evaluated as an expression of pain. The behavior of an organism has been proposed as a tool for measuring/evaluating nociception and pain in nonmammalian animals, as the observed responses in mammals are different (analgesic self-administration, autotomy, conditioned place aversion, gait/weight bearing disturbance, grip/bite force, scratching/licking/biting, guarding, abnormal positioning, paw lifting/flinching/shaking, hypolocomotion, dysorexia/anorexia and weight loss, inattention to novel stimuli, and ultrasonic vocalization [45]).

In the past, the behavioral tests adapted to reptiles were reduced to the classic nociceptive tests of reflex response to noxious stimuli such as thermal threshold or electrostimulation [41,51,52,53,54,55,56,57,58,59,60]. The results were inconclusive or even unexpected, such as the enhancement of the algic response to formalin and capsaicin after treatment with amitriptyline in Speke’s hinge-back tortoise *Klinysis spekii* [61]. Chemical stimulation tests (formalin, capsaicin) that can induce more persistent pain have been used as pain assessment methods in reptiles [62,63].

Experimental models have not been extensively validated for their discriminatory abilities in reptiles. Starting from an evolutionary point of view, when we consider pain and noxious behavior across species, families, and phyla, the first concern is precisely about the scarce ecological validity of the intense thermal stimulus and electrostimulation used in current practice in research [14]. Among these, thermal testing is particularly puzzling and lends itself to further misinterpretations in heterothermic animals such as reptiles. It uses the thermal withdrawal reflex to assess pain response, but reptiles are heterothermic; thus, several questions have been raised about whether to use it as analgesic efficacy index [64]: Does the thermal threshold change between the warming and cooling periods? Could the physiological search for heat lead to an extension of the thermal latency time regardless of the drugs administered? Could reptiles adapted to extreme environmental situations (e.g., desert) have greater tolerance than species of temperate climates? Why do captive reptiles get burned so often? This latter circumstance suggests that heat may not be perceived in all reptile species as harmful enough to elicit a withdrawal response to avoid thermally induced tissue damage [36]. It has been hypothesized that thermoreceptors and nociceptors are different and sensitive to different thresholds [65]. To further complicate the interpretation of the results of pain tests in reptiles, there is also the possibility that, when the stimulus is applied to a particular area of the body (legs for saurians and testudinates, abdomen for ophids), the localization and density of thermoreceptors may vary in different areas of the body [9] due to the different anatomical, physiological, and behavioral adaptations for thermoregulation of an entire class of heterotherm vertebrates. However, some authors argue the applicability of the thermal test to reptiles, which would manifest withdrawal reactions indistinguishable from those of mammals [66]; considering the current poor understanding of the perception of heat by reptiles, the question remains open.

There are behaviors that can be observed in snakes, lizards, and turtles in the course of any disease. When dealing with pets, the owner’s pain and anxiety relief should not be underestimated, as owners may be more aware of what describes normal behavior for that pet [67], while the assessment is certainly more difficult when it comes to wild species such as sea turtles. Many of the nociceptive tests described in mammals, based on the recognition of abnormal behaviors, are difficult to apply to wild reptiles, and they require a deep knowledge of physiology, behavior, and species-specific adaptations of the observed species to discriminate the manifestation of behaviors other than normal and contextualizing them to the specific environment where the observations take place [9]. In wild and shy animals such as sea turtles, it remains difficult to understand whether any behavioral alterations are really manifestations of pain, sickness, or fear/distress or what should be the “normal” behavior considering the captive conditions of the animal. To do this, a species-specific and context-specific behavioral ethogram should be developed. A study on the possible analgesic effect of morphine and butorphanol prepared an ethogram for *Trachemys scripta* based on feeding behavior, willingness to swim, and respiratory rate, evaluated before and after surgery, enrolling for the test only animals that adapted to the experimental conditions before the test [68], but this model of pain is predictable, while a rescued sea turtle is generally in difficulty due to a problem, also presumably painful. Therefore, it is difficult to apply a “before” and “after” behavioral alteration.

An approach has been proposed [9,35] to the assessment of pain in reptiles that is divided into several parameters, some of which are applicable to reptiles kept in captivity but certainly not to wild individuals:

- behavioral: they must include species considerations, relating to attitude (predator/prey, diurnal/nocturnal, arboreal/terrestrial/aquatic/fossorial) and individual parameters (ecdysis stage, hibernation, sociability, intercurrent illnesses)
- environmental: they must include the preferred optimal temperature zone (POTZ) and consequent metabolic rate, enclosure setting, presence of the observer
- locomotion: posture, gait, excessive scratching, or flicking foot, tail, or affected area are quite unapplicable to chelonians
- exaggerated fight response: actually present in sea turtles in good condition as a reactive mechanism to handling
- appetite: when dealing with wildlife, adaptation to captive diet must be considered, and some days of anorexia may not be related to pain
- color alterations: useful in saurian species capable of color changes, but not chelonians and snakes
- eyes (open/closed)
- respiratory model (difficult to evaluate in aquatic chelonians)
- physiological: they can also be altered only by the test conditions (excitement, fear for wild animals)
- response to palpation: not very reliable in reptiles in general, not accustomed to contact with other individuals, and unpractical for wild species, particularly for chelonians due to the carapace.

Behavioral models are even more difficult to apply to the order Chelonia because of the physical conformation (presence of the carapace) which makes it impossible to assume abnormal postures related to pain, otherwise possible in other species. In addition, many of the pain models proposed for reptiles are rather controversial, and the ambiguous response manifested by different species of chelonians to opioids must often be attributed to the inconsistencies in the quality of the experimental design (pain model used) and the strength of the conclusions (evaluation criteria) [69].

Emphasizing that, in reptiles in general (and even more in little young sea turtles because of small size and less thermal inertia), observations must be conducted with animals kept in the POTZ, it is easy to deduce how difficult it is to identify and quantify pain in this animal class, considering that many behaviors attributed to suffering in other species may already be the expression of a response to a situation of discomfort or fear. Furthermore, reptiles and other nondomestic species are reluctant to exhibit behaviors that are clearly associated with pain, to decrease the likelihood of being recognized as sick by predators, and, considering that immobility is a common survival tactic for prey species, it is more difficult to assess whether pain is present in these subjects.

In addition to behavioral models, the survey of physiological parameters (heart rate, respiratory rate, plasma levels of catecholamines and cortisol) has also been proposed as a tool for the relief of pain-induced changes. In a study on the use of meloxicam and butorphanol in pre-emptive analgesia in ball python *Pito regius*, these parameters did not show any difference/clinical efficacy between the treated group and the control [70].

## 4. Pain Rating Scales

Veterinarians and researchers involved in animal care and welfare recognize the need for sensitive methods of assessing animal pain, because pain is a subjective experience rather than an objectively quantifiable physiological response; therefore, its assessment can be very difficult. The experience of pain is highly variable between individuals, even if identical stimuli are applied under equivalent environmental conditions. Furthermore, the experience of pain and its behavioral consequences varies considerably between species, and even the individual’s behavior is the result of complex relationships between its internal and external environments [71].

Systematic attempts to objectify pain assessment in an objective and repeatable way have also led in the past to the development of pain rating scales, based on selected behavioral pain indicators; some of them also include the assessment of some physiological parameters. Furthermore, grimace scores have been added to immediately detect and assess the signs of pain, first in laboratory animals [72]. There are many pain scales and questionnaires that have been developed to capture and quantify the individual pain experience for verbal humans, and some have been adapted to nonverbal species in veterinary medicine. Most pain scales are quite context-specific and only suitable for assessing a particular type of pain (i.e., acute or chronic), and they are frequently species-specific. However, their main bias is linked to their basis on the subjective evaluation of parameters whose correlation with other behavioral or physiological indicators of pain and/or stress have not been fully confirmed and whose rating is affected by the subjectivity of the observer, with inter- and intra-observer variability, because of which the repeatability of the observation loses reliability [70,73].

## 5. Analgesic Therapy

Keeping in mind that, when we talk about “reptiles”, we are actually defining a whole class, with four distinct orders and an extreme variety of evolutionary adaptations, it is easy to deduce how dangerous it can be to generalize and apply recommendations to the whole group. For the same reason, it is unthinkable to extrapolate from one species or order (e.g., *Sauria*) and apply it to a completely different one (e.g., *Chelonia*) only on the basis that both are “reptiles”.

A careful and adequate analgesic therapeutic plan should involve the choice of the most suitable molecule for the type of pain to be controlled, the definition of the most adequate route of administration (especially in prolonged repeated treatments), and the most suitable dose, all followed by careful monitoring the patient’s response, as well as the ability to intervene promptly to counteract any unforeseen unwanted side-effects [64]. Borrowing a posology from other species is always dangerous if the peculiarity of the present one is not known, and, when available, it is always good to refer to pharmacokinetic and pharmacodynamic studies. In this regard, for example, the literature on NSAIDs is quite abundant with respect to pure pharmacokinetic studies (see below), and few cases integrate PK and PD, where the pharmacodynamic test used is always the thermal threshold, with all the doubts already expressed [74,75]. In all cases, however, their results must be interpreted with critical evaluation. Most of the times, the tested dosage was chosen empirically or inferred from other species; with no data available on the clinical efficacy in the single species (back to the bias of the type of test chosen for the evaluation of the effectiveness and difficulty of the pain assessment), the only considerations that can consistently be made are the differences with other species, the plasma concentrations achieved, and the other kinetics parameters, advancing speculations on the probability of equal efficacy in the reptile species under examination, while nothing can be inferred about what would actually happen in terms of clinical efficacy and safety.

### 5.1. Systemic Analgesia

#### 5.1.1. Opioids and Opioid-Like Drugs

Opioids are a group of natural or synthetic substances capable of binding to specific receptors, thus inducing different effects, with the most important and desirable in clinical practice being analgesia. Their classification as μ, κ, and δ agonists/antagonists is based on the identification of different receptor subtypes:

- The μ receptors, to which β-endorphins bind preferably, are involved in causing supraspinal analgesia, respiratory depression, hypothermia, bradycardia, mydriasis or myosis, euphoria, sedation, and physical dependence
- The κ receptors, to which dynorphins bind mainly, are responsible for spinal analgesia, miosis, modest degree of sedation, dysphoria, a certain degree of respiratory depression, and vasomotor stimulating effects
- The δ receptors are activated by the enkephalins, thus producing excitation, hyperkinesis, euphoria, hallucinations, peripheral analgesia, respiratory depression, and mydriasis.

Synthetic opioids act as agonists toward some receptor types and as antagonists or partial agonists toward others, justifying the complex pharmacological picture that derives from their administration.

It has been documented that the opioid receptor gene family is well conserved in all vertebrates (see above and [76]), and both proenkephalin-derived peptides and opioid μ, κ, and δ receptors have been identified in *Trachemys scripta elegans* [24,77]. Clinical efficacy studies in different reptile species are available [41,51,52,53,54,55,56,57,58,59,60,62,78,79], as are pharmacokinetic studies [80,81,82,83]. The most frequently tested opiate was butorphanol, which, despite being frequently used in clinical practice [39], was found to be ineffective in the treatment of pain in these studies. The kinetics of buprenorphine have been studied [80], but the molecule was found to be non-analgesic in studies conducted with the thermal test [54] and electrostimulation [51]. Better results appear to be derived from morphine, especially when the pain test was the formalin test [84], but respiratory depression was noted at high doses (hard to discriminate analgesia from sedation). Although there is also controversy in the use of the pseudo-opioid tramadol in pets, due to the need for its hepatic activation in *O*-desmethyltramadol, (which is produced in the loggerhead sea turtle *Caretta caretta* [85]), this drug has also been tested in red-eared slider *Trachemys scripta elegans* despite the inconvenience of oral administration [53]. The relative ease of transdermal administration of fentanyl via patch (despite the scales of the skin surface of reptiles) has attracted some researchers, who have demonstrated its systemic uptake via this route in ball python *Python regius* and corn snake *Elaphe guttata* [86], as well as the plasma kinetics in ball python [56] and prehensile-tailed skink [82], without being able to conclude whether plasma concentrations can be effective in clinical use as an analgesic drug, having found no efficacy of the thermal test.

In fact, all clinical efficacy studies should be carefully evaluated for the experimental design (particularly the pain test chosen for the evaluation of the effect) and the strength of the conclusions, where the only legitimate ones are those directed toward the variability of the role of receptors for opioids within the order Reptilia [64]. The application of noncritically evaluated data can have dire consequences, as reported by Sladky regarding the use of butorphanol [67]. In the cited literature, the author pointed out that data of their studies of butorphanol in corn snake were “too variable to make a firm decision” [52]; however, despite this, some clinicians used it in debilitated snakes, and “fatal consequences occurred in some cases”.

#### 5.1.2. NSAIDs—Nonsteroidal Anti–Inflammatory Drugs

Numerous molecules belonging to this class of drugs, traditionally classified on the basis of their chemical structure, are currently among the most used for the management of acute and chronic pain and inflammation in human and veterinary medicine. Although extremely heterogeneous from a chemical point of view, all these compounds share a common mechanism of action that justifies their grouping in the same pharmacological class, and the therapeutic and side-effects related to these drugs can be attributed to it. Nonetheless, important qualitative and quantitative differences in pharmacodynamic, pharmacokinetic, and toxicological behavior depend on the different structural characteristics. Almost all the t-NSAIDs (traditional) are commonly referred to as cyclooxygenase synthase (COX) inhibitors. All these molecules, in fact, inhibit in vitro and in vivo the two isoforms of COX (constitutive COX-1 and induced COX-2) with equal power, and, at the concentrations that are achieved in the blood and tissues following the administration of therapeutic doses, they inhibit the activity of both isoenzymes almost completely (90–100%). Some molecules more recently introduced in human and veterinary clinical practice (nimesulide, meloxicam, carprofen, etodolac, eltenac) exhibit a greater selectivity toward COX-2. These compounds, in fact, are in vitro 10 to 20 times more potent in inhibiting the activity of COX-2 than COX-1 and are, therefore, indicated as preferential inhibitors of COX-2, a behavior that explains the best tolerability profile shown by these drugs in clinical practice [87]. Molecules with greater selectivity for COX-2, the “coxibs”, are already available for humans and pets, but their use in reptiles is still to come.

The role of cyclooxygenase in the pathophysiology of pain and inflammation in reptiles has not yet been defined; however, practitioners continue to use them, reporting positive effects [37] even without the support of scientific data. The only reports available are related to pharmacokinetic studies [88,89,90,91], including those specifically in sea turtle [81,90,91,92,93,94,95,96], from which nothing can be inferred, as plasma concentrations do not correlate with clinical efficacy for this class of drugs. There is only one study that aimed to evaluate the analgesic efficacy of meloxicam in ball python for postoperative pain control, and the result was an apparent ineffectiveness [70].

COX-1 and COX-2 were both found to be constitutively expressed in healthy and traumatized tissues of eastern box turtle *Terrapene carolina carolina*, although the inflammatory stimulus upregulates the expression of COX-2; hence, NSAIDs active on both isoforms are expected to be more effective if compared to selective COX-2 [97], while, in ball python *Python regius* the traumatized tissues expressed more COX-1 in a thermal noxious stimulus model [98], and the use of selective COX-2 inhibitors again seems pointless. Despite this, most of the studies (kinetic and clinical) on NSAIDs have focused on meloxicam, a preferential COX-2 inhibitor.

Overall, all available studies on the applicability of NSAIDs in sea turtles are pharmacokinetic studies of single doses, with no attempt to assess clinical efficacy or safety. Few studies have explored multiple administration, such as for ketoprofen in loggerhead turtle *Caretta caretta* [99] and meloxicam in Kemp’s Ridley (*Lepidochelys kempii*) and green (*Chelonia mydas*) turtles [100].

Considering the possible onset of biochemical and hematological alterations (albeit defined as mild) following repeated dosing protocols of meloxicam and carprofen in green iguana [101,102], the possibility that meloxicam has an enterohepatic circulation and urinary reabsorption [100], and how little we know of the possible onset of toxic effects similar to those reported in mammals (GI irritation, platelet aggregation inhibition, renal impairment), the principle of caution seems mandatory [64]. Furthermore, the necessity for studies on every single species and the danger of generalization have been evidenced by studies in three sea turtle species (*Caretta caretta, Lepidochelys kempii*, and *Chelonia mydas*), where significant differences were found in the pharmacokinetic parameters of meloxicam, and the hypothetic therapeutic plasma level was inferred from the concentrations that show anti-inflammatory activity in humans, as data on pharmacodynamic and clinical efficacy of meloxicam in sea turtle are not currently available [82,100].

### 5.2. Local Analgesia

The term “local anesthetics” includes a heterogeneous pharmacological class unified by a similar mechanism of action, based on the transient and reversible interruption of nerve conduction at the site where these molecules are applied. Among the numerous substances with this property, different physicochemical characteristics and other factors affect their clinical activity and toxicity. In reptiles, these molecules have been used as loco-regional anesthetics for minor surgical interventions [2,4], but they can also be used as analgesics. However, recent studies have shown that the use of these drugs has to be limited to the peri-operative period and the animal’s hospitalization, due to the short duration of the analgesic effect and the motor paralysis they cause [36]. Local anesthetics are thought to have retained their efficacy across different vertebrate taxa due to their peripheral motor and sensory nerve conduction-blocking mechanism. Lidocaine, bupivacaine, or mepivacaine are expected to maintain only local effects without relevant systemic actions, but no toxic dose data are available for reptiles. For use as branch blockers, mepivacaine and lidocaine were tested; the former proved to be effective as a mandibular nerve blocker in the American alligator *Alligator misissippiensis*, Yacare caiman *Caiman yacare*, and dwarf crocodile *Osteolaemus tetraspis* [103], while the latter proved to be effective as an analgesic of the prefemoral fossa in the Chinese box turtle *Cuora flavomarginata* subjected to coelioscopy, in which it was found to be ineffective [104]. For spinal analgesia, the effective application of intrathecal lidocaine, bupivacaine, and morphine to red-eared slider turtle *Trachemis scripta elegans* [105], lidocaine to hybrid Galapagos tortoise (*Geochelone nigra*) [106], and the α_2_-blocking action of intrathecal clonidine to marsh terrapin *Pelomedusa subrufa* [107], successfully antagonized by yohimbine, has been described.

### 5.3. Multimodal Analgesia

The use of different classes of analgesic drugs combined to obtain the maximum effect of attenuation/elimination of the pain perception involves the synergistic application of opioids that act centrally and peripherally to alter the physiological response to pain, NSAIDs that act at the tissue level to the control of inflammatory pain, and/or local anesthetics that block the transmission of the pain signal from the periphery to the NCS. When used together, these drug associations could have the best choice to manage pain, especially when they are used preemptively, before a programmed painful procedure (e.g., surgery) is started [2,3,4] and in debilitated patients [12]. Currently, no studies have been carried out on reptiles in this field, although the advantages of this association have already been exploited in veterinary medicine for mammals [2,3,4].

## 6. Conclusions

When choosing a therapeutic plan for a wild species (in this case, for a sea turtle), it must be borne in mind that all commercially available drugs are registered for use in humans and/or in the most common domestic species; therefore, they present pharmaceutical forms authorized and formulated for this purpose. Consequently, great attention should be paid to the posology as they all will be off label when used in reptiles. On the other hand, the term “reptiles” identifies such a variety of different orders/families/species, with many different anatomical, physiological, and metabolic adaptations to different ecological niches, whereby generalization within the same Reptilia class can involve serious risks of therapeutic failure at best and lethal outcome at worst. In this respect, the greatest care must be paid when applying protocols available in many formularies [2,4,12,62,108] only based on having been established or tested in a “reptile” species, without verifying from bibliographic references which was the species and, above all, the result.

When can an analgesic drug be used as a pain reliever in sea turtle? This is a question that currently does not have a single and exhaustive answer. Reptiles are a very particular and diversified class of animals that have developed peculiar mechanisms for the management of body temperature and metabolism and do possess the anatomical and physiological structures to “feel” pain beyond the proprioceptive perception. For this reason, despite the difficulties inherent in the study of reptile pain, clinicians should assume that pain is present in reptiles as it would be in species for which more nociception data are available; therefore, a painkiller should be administered in all those cases in which it would be administered to a mammal. However, considering current partial knowledge and understanding of their physiology, and despite the desire to have a tool for the correct management of post-traumatic or post-operative pain in sea turtles, the truth is that the available data are fragmented, dangerous to generalize, related to single aspects or species, or obtained with methodologies whose reliability in heterothermic species is still under discussion. Therefore, pending further research developments that allow an objective evaluation, devoid of personal bias and beliefs, the invitation to the operators involved in sea turtles rescue and rehabilitation is not to improvise or to infer posology and therapeutic protocols from other species. A special effort should be made in choosing the safer analgesic therapy after considering the pros and the cons of drugs that have adequate scientific backgrounds, in order not to add the iatrogenic threat to the many to which these ancient animals are already exposed.
